# Personality Traits vs. Sports Classes of Polish Representatives in Junior Sports Acrobatics

**DOI:** 10.3390/sports11100194

**Published:** 2023-10-06

**Authors:** Paweł Piepiora, Adrianna Naczyńska

**Affiliations:** Faculty of Physical Education and Sports, Wroclaw University of Health and Sport Sciences, 51-612 Wrocław, Poland

**Keywords:** sports psychology, Big Five, gymnastics, karate, high school students

## Abstract

The aim of this paper was to determine the influence of adolescence, training discipline, and training regime on the personality formation of adolescent sports acrobats. Therefore, the purpose of this paper was to study the relationship between the personality traits and the sports classes of Polish junior sports acrobatics representatives. The respondents (N = 90) were juniors aged 18–19 and were divided into three samples: (1) n = 30 Polish representatives in sports acrobatics with a first or master sports class; (2) n = 30 kata athletes from Kyokushin karate and Shotokan karate as a reference sample from another sports discipline with gymnastic movement expression; (3) n = 30 high school students as a reference sample of non-athletic persons. The Big Five model was used, and the NEO-FFI personality questionnaire was used as a research tool. For the analyses of basic descriptive statistics, the Kruskal–Wallis test and Mann–Whitney test were performed to determine statistical significance (α = 0.05). It was noted that all athletes (the first and second samples) had personality traits at the same levels: low neuroticism, high extraversion, moderate openness to experience and agreeableness, and high conscientiousness in relation to non-athletes. In contrast, there were differences among the subjects in personality traits, except for agreeableness. Among the sports acrobats, master-class athletes showed lower neuroticism, greater openness to experience, lower agreeableness, and greater conscientiousness in relation to first-class athletes. It was found that there were differences between the personality traits and sports classes of junior sports acrobatics representatives in Poland in the four Big Five dimensions. But in general, sports acrobats and karate athletes had personality traits at similar levels, and at the same time, different from non-training people.

## 1. Introduction

Sports acrobatics, also known as acrobatic gymnastics, is a sport that involves the presentation of gymnastic circuits of great difficulty [1]. It includes exercises for men and women in pairs or in groups, as well as individual or synchronized exercises using trampolines and gymnastics tracks [2]. It is important to note that each of the categories of sports acrobatics (baton jumping; path jumping; team exercises: static, dynamic, combined; mini doubles) is characterized by its typical acrobatic circuits [3].

Sports acrobatics is a good foundation for development in any other sport. That is why it is so popular among children [4]. It is an excellent method of preparation and a supplement to practice in other sports. Thus, sports acrobatics has a positive effect on improving physical fitness and motor skills [5]. In addition, according to coaches of sports acrobatics, this specific physical activity contributes to proper physical and mental development in children [6]. Young sports acrobats learn to perform gymnastic elements that allow them to release the energy accumulated in their bodies, as well as stretching exercises that lead to relaxation [7]. In addition, sports acrobats, by mastering new gymnastic circuits, overcome their own fears and limitations and learn self-discipline [8]. On the other hand, the discipline and training regime are the main reason why teenage sports acrobats give up training [9]. The monotony of multiple repetitions of gymnastic circuits in pursuit of perfection often loses out to the problems of the adolescent period of sports acrobats [10]. Ultimately, this results in the low popularity of sports acrobatics among adolescents [11].

The adolescent period begins around the age of 11 and lasts until the age of 19. Furthermore, the age of 16 is assumed to be the most significant as conflicts with peers increase during this time [12]. This is also when the greatest increase in neuroticism occurs. A teenager may experience anxiety, stress, and tension more intensely during the adolescent period. Excessive worry and hypersensitivity to stressful situations are also noted [13]. The adolescent period ends with the conscious formation of one’s own identity. Therefore, in this period, the support of the adolescent’s family and circle of loved ones is of great importance in undertaking sports activities as well as other life issues [14].

The problems and dilemmas described above undoubtedly affect the formation of athletes’ personalities [15]. It is important to remember that a humans’ personalities at birth are formed by genetic factors. After that, their functioning in social systems influences their personality. Therefore, considering a person’s life from birth to death, it is assumed that human personality is shaped by the environment. It is shaped by experiences, contacts with people significant to the individual, social roles played by the person, as well as recurrent or other events that are exceptionally strong [16]. It is worth noting that the topic of personality in sports is still relevant, and previous research results on this topic in the field of sports psychology are inconclusive [17]. Therefore, the purpose of this paper was to verify the differences between the personality traits and sports classes of juniors training in high-level sports acrobatics who are part of the Polish National Team. A research question was formulated: do junior sports acrobats of the master class differ from junior sports acrobats of the first class? It was decided to verify the hypothesis that sport level at the junior age significantly differentiates the personality traits of sports acrobats.

## 2. Methodology

### 2.1. Research Subjects

This study involved 90 people aged 18–19, who were divided into three samples. The first sample consisted of representatives of Poland in sports acrobatics (n = 30; including 21 women and 9 men) who are associated with the Polish Gymnastics Federation and have a first sports class or master sports class. They competed in the following competitions: path jumping and static, dynamic, and combined team exercises. Data on the sports classes of the surveyed people were available only for the first sample. Since sports acrobatics consists of presenting gymnastic sequences, it was decided that the second sample must be athletes from another discipline who also specialize in expressive motor routines. Therefore, the second sample—a reference sample of professional athletes—consisted of kata competitors from Kyokushin karate and Shotokan karate (n = 30, including 11 women and 19 men) who are associated with the Polish Karate Federation. Kata are formal circuits consisting of a sequence of coordinated movements that are performed in a fixed order and to a specific rhythm. Each kata presents an imaginary fight against attackers. The third sample—a reference sample of people who do not train sports—included students (n = 30, including 15 women and 15 men) from the General Secondary School in the Complex of Secondary Schools and Sports Championship in Szklarska Poręba. Their selection was determined by their availability as peers of the respondents from the first and second samples.

### 2.2. Research Tool

The Big Five model was used in this research. It is a five-factor model of personality that assumes five dimensions in the theoretical construction of personality: neuroticism, extraversion, openness to experience, agreeableness, and conscientiousness [18]. Each of these dimensions has an opposite pole (neuroticism vs. emotional constancy; extraversion vs. introversion; low vs. high openness to experience; agreeableness vs. antagonism; conscientiousness vs. undirected), so they allow for a full description of human personality, and the intensity of individual dimensions is rated on a scale from 1 to 10 stens [19]. The Big Five is the most reliable and proven theory of personality traits. It assumes that the dimensions of personality exist and are important in adapting an individual to their environment. They are also permanent and universal, that is, independent of race, gender, and culture. Moreover, they are biologically conditioned because they are characterized by a high degree of heritability [20]. To determine personality traits using the Big Five model, the NEO-FFI personality questionnaire was used, which is a shortened version of the NEO-PI personality inventory and is widely used for the purposes of theoretical and applied sports psychology. Measurement reliability using the NEO-FFI scales was lower than for the NEO-PI scales, but still satisfactory: Cronbach’s alpha = 0.86 neuroticism, 0.77 extraversion, 0.73 openness to experience, 0.68 agreeableness, 0.81 conscientiousness. In contrast, for theoretical relevance, the correlations ranged from 0.56 openness to experience to 0.62 neuroticism, and were significantly higher than the correlations of uncorrelated traits. The NEO-FFI consists of 60 self-descriptive statements whose truthfulness in relation to oneself is assessed by the respondent on a five-point scale. There are 12 statements in each dimension. NEO-FFI has adopted standards that refer to gender and age in the following ranges: 15–19 years, 20–29 years, 30–39 years, 40–49 years, 50–80 years [21].

### 2.3. Procedure

The spatial scope of this research encompassed the territory of the Republic of Poland, while the time span was one year and included the period from May 2022 to April 2023. The questioning of athletes was carried out during sports training camps, and the questioning of non-training participants was carried out during classes with a school pedagogue. The criterion for the inclusion of the subjects was a voluntary and written willingness to participate in the cognitive experiment. All respondents agreed to the use of the results obtained for scientific and research purposes by the Wroclaw University of Health and Sport Sciences with full anonymity. Furthermore, all subjects received instructions to complete the NEO-FFI prior to completing the questionnaire. The working time of the subjects for the NEO-FFI was up to 60 min. Due to the ages of the surveyed persons, the NEO-FFI norms for ages of 15–19 were adopted. The project was implemented based on the positive opinion of the Senate Committee for Research Ethics at Wroclaw University of Health and Sport Sciences, number 20/2019.

### 2.4. Statistical Analysis

Statistical analyses were performed using IBM SPSS Statistics 27.0 software, including the analysis of basic descriptive statistics using the Kruskal–Wallis test and Mann–Whitney test. The level of statistical significance was α = 0.05. Based on the obtained data, a sensitivity analysis of the statistical test was carried out before the start to determine the required effect size. With a sample size of N = 90, a level of α = 0.05, and a statistical power of 95%, it was possible to detect an effect of ηp^2^ = 0.15 (Cohen’s f = 0.42).

## 3. Results

### 3.1. Basic Descriptive Statistics

In the first step of the analysis, basic descriptive statistics were determined along with the interpretation of the average intensity of features based on the proposed standards. The analyses used the results converted into stens, referring to Polish standards. The basic descriptive statistics, together with tests of the normality of the Kolmogorov–Smirnov distribution, are presented in Table 1. The mean and median of individual personality traits along with the interpretation of the average intensity of a given trait are presented in Table 2.

As part of the analysis of the consistency of the distribution of variables with a normal distribution, it was observed that the distribution of variables for individual features broken down into the practiced sports disciplines differs from the normal distribution. Therefore, in the next step of the analyses, comparisons were made using a non-parametric statistical test.

### 3.2. Personality Traits and Sports Disciplines

In the next step of the analysis, we verified whether sports acrobats differed in the intensity of their individual personality traits from karatekas and non-training people. For this purpose, the non-parametric Kruskal–Wallis test was performed. Detailed results are presented in Table 3, while pairwise comparisons including post hoc tests with Bonferroni correction are presented in Table 4.

The analysis showed that there were differences between the subjects in their levels of neuroticism, extraversion, openness to experience, and conscientiousness. Sports acrobats and karate athletes did not differ in the intensity of the above-mentioned traits. But athletes from both sports showed a lower intensity of neuroticism and a higher intensity of extraversion, openness to experience, and conscientiousness in relation to non-athletes.

### 3.3. Personality Traits and Sports Classes

Next, we examined whether the sports acrobats differed from each other in the intensity of their personality traits depending on their sports class. For this purpose. It was observed that among sports acrobats, master-class athletes were characterized by a lower intensity of neuroticism than first-class athletes. A higher intensity of openness to experience was also observed among master-class athletes. Interestingly, master-class athletes were characterized by lower levels of agreeableness. In addition, they were observed to have a higher intensity of conscientiousness than athletes of the first sports class. No differences were observed between athletes in the intensity of extraversion. Detailed results of the analysis are summarized in Table 5.

## 4. Discussion

The obtained study results provide cognitively interesting data. The representatives of Poland in sports acrobatics at a junior age differ in their personality traits by sports class. Master-class sports acrobats are characterized by lower neuroticism, higher openness to experience, lower agreeableness, and higher conscientiousness in relation to first-class sports acrobats. This means that master-class athletes are more emotionally stable, calm, balanced, and relaxed; handle difficult life situations well; are satisfied with themselves; have a sense of security; and stressful situations do not make them nervous and do not throw them off balance. They are also more curious about the external and internal world, find pleasure in inventing new things, recognize unconventional values, experience emotions more intensely, seek new experiences and sensations for themselves, and have a vivid imagination. In addition, they are more self-centered, skeptical about the intentions held by others, vulgar, suspicious, judgmental, have a competitive rather than cooperative attitude, tend to manipulate others, and are reluctant to cooperate. Also, they have more ambitious goals, stronger willpower, strong determination, and are more meticulous, punctual, reliable, disciplined, hardworking, and ambitious [21]. This shows that personality traits play an important role in master-level sports acrobats. Therefore, important value should be given to personality traits in addition to the psychophysical training of athletes [22]. The personality traits of sports acrobats may be one of the prerequisites for entering a higher level of sports.

In addition, it is significant that sports acrobats and karate athletes have the discussed personality traits at the same levels, which differs them from non-trainers. Athletes are characterized by low levels of neuroticism, high levels of extraversion, moderate levels of openness to experience and agreeableness, and high levels of conscientiousness in relation to non-athletes, who have all these traits at moderate levels. This indicates that personality traits play an important role in the daily functioning of individuals [23,24]. People who manifest low levels of neuroticism, high levels of extraversion, moderate levels of openness to experience and agreeableness, and high levels of conscientiousness tend to participate in sports activities [25].

The question of the raw scores of the studied athletes is also important. Sports acrobats do not differ in their severity of neuroticism from karate athletes, but both groups of athletes have a lower severity of neuroticism than non-athletes. Sports acrobats also do not differ in their intensity of extraversion from karate athletes, but both groups of athletes have a higher intensity of extraversion than non-athletes. Sports acrobats also do not differ in their intensity of openness to experience from karate athletes, but both groups of athletes have a higher intensity of openness to experience than non-trainees. In contrast, there are no differences between sports acrobats, karate athletes, and non-trainers in their intensity of agreeableness. In addition, sports acrobats do not differ in their intensity of conscientiousness from karate athletes and both groups of athletes have higher intensity of conscientiousness than non-athletes. This indicates that personality traits are shaped by the specifics of the sport in which athletes are trained [26]. The human personality is formed by the environment, and sports are part of this environment [27]. Therefore, it is important to recognize the influence of the specifics of the sports activity undertaken on the formation of athletes’ personalities [28,29].

Therefore, the distribution of personality traits influences taking up sports activities, which is evident in the comparisons between athletes and non-trainers. In addition, training in a selected sports activity shapes the personality of athletes, which is evident in the comparisons between sports acrobats and karate athletes, and between athletes and non-trainers. And most importantly, the personality traits of sports acrobats have different intensities in relation to sports classes. This results in a knowledge of the personality traits of Poland’s top-level junior sports acrobatics representatives. But an open question remains regarding to what extent the already-formed personality traits of the sports acrobats had an impact on their achievement of championship level, and to what extent specific training in sports acrobatics was significant in the formation of the recorded intensities of their personality traits? There was no detailed knowledge of the history of disorders [30] and diseases [31] among the subjects, so objective verification is not possible. The advantages of training sports acrobatics are known [32] and valued [33,34], but in this case, it would be a one-sided decision.

### 4.1. Study Limitations

The present study is limited to Polish citizens aged 18–19 years and specific sports: sports acrobatics and karate (kata athletes from Kyokushin karate and Shotokan karate). Furthermore, there are no data on the respondents from before and after the COVID-19 pandemic, which further limits the study outcome temporally. Therefore, it was concluded that the present study is limited by population, culture, and time. With this in mind, the results obtained from the juniors can be used for comparisons with athletes from Central Europe.

### 4.2. Further Research Possibilities

It would be advisable to continue studying the topic undertaken while considering athletes of all individual sports, combat sports, and team sports. Also, it would be interesting to conduct research work on an international scale in all age categories.

### 4.3. Practical Implications

The results obtained show differences in the personality traits of junior athletes between master-class and first-class sports acrobats. It was observed that, in addition to sports training and physical skills, the personality traits of the athletes also play an important role in the differences between sports levels. This confirms the validity of implementing mental training for athletes. Therefore, comprehensive training should be carried out for athletes in the areas of motor, technical, tactical, and mental preparation.

## 5. Conclusions

There are differences between the personality traits and sports classes of the representatives of Poland’s junior sports acrobats. A higher sports class is associated with lower neuroticism, higher openness to experience, lower agreeableness, and higher conscientiousness in sports acrobats. However, the athletes studied (sports acrobats and karate athletes) do not differ in the discussed personality traits, and their personality profiles are typical of athletes.

## Figures and Tables

**Table 1:** Basic descriptive statistics obtained from test of normality of distribution.

	*M*	*Mdn*	*SD*	*Sk.*	*Kurt.*	*Min.*	*Maks.*	*K-S*	*p*
Neuroticism	3.66	3.50	1.15	0.40	−0.60	2.00	6.00	0.21	<0.001
Extraversion	6.88	7.00	0.95	−0.40	−0.77	5.00	8.00	0.22	<0.001
Openness to experience	5.60	5.00	0.83	0.27	−0.68	4.00	7.00	0.28	<0.001
Agreeableness	5.50	5.00	0.59	0.34	−0.48	4.00	7.00	0.33	<0.001
Conscientiousness	7.22	7.00	0.98	0.27	−0.96	6.00	9.00	0.20	<0.001

*M*—mean; *Mdn*—median; *SD*—standard deviation; *Sk*—skewness; *Kurt*—kurtosis; *Min*—the lowest value of the set; *Max*—the highest value of the set; *K-S*—Kolmogorov–Smirnov test; *p*—significance level.

**Table 2:** Personality traits and the practiced sports disciplines.

Discipline	*Acrobatics*			*Karate*			*Non-Trainers*		
	*M*	*Mdn*	*Reading*	*M*	*Mdn*	*Reading*	*M*	*Mdn*	*Reading*
Neuroticism	2.83	3.00	Low	3.20	3.00	Low	4.93	5.00	Moderate
Extraversion	7.30	7.00	High	7.60	8.00	High	5.73	6.00	Moderate
Openness to experience	6.17	6.00	Moderate	5.80	6.00	Moderate	4.83	5.00	Moderate
Agreeableness	5.57	6.00	Moderate	5.60	5.50	Moderate	5.33	5.00	Moderate
Conscientiousness	8.00	8.00	High	7.50	7.50	High	6.17	6.00	Moderate

*M*—mean; *Mdn*—median.

**Table 3:** Comparison of personality traits of athletes and non-trainers.

	*Acrobats*		*Karatekas*		*Non-Trainers*			
*Variables*	*Mdn*	*M*_*rank*_	*Mdn*	*M*_*rank*_	*Mdn*	*M*_*rank*_	*H*	*p*
Neuroticism	3.00	27.25	3.00	36.00	5.00	73.25	56.27	<0.001
Extraversion	7.00	56.00	8.00	65.00	6.00	15.50	67.28	<0.001
Openness to experience	6.00	61.63	6.00	52.70	5.00	22.17	43.41	<0.001
Agreeableness	6.00	48.87	5.50	48.15	5.00	39.48	3.08	0.214
Conscientiousness	8.00	64.67	7.50	54.25	6.00	17.58	58.52	<0.001

*Mdn*—median; *M_rank_*—mean rank; *H*—Kruskal–Wallis test statistics; *p*—significance level.

**Table 4:** Results of pairwise comparisons of the intensity distributions of athletes’ personality traits.

	Neuroticism	Extraversion	Openness to Experience	Agreeableness	Conscientiousness
Acrobatics–karate	−8.75	−9.00	8.93	0.72	10.42
Acrobatics–non-trainers	−46.00 ^b^	40.50 ^b^	39.47 ^b^	9.38	47.08 ^b^
Karate–non-trainers	−37.25 ^b^	49.50 ^b^	30.53 ^b^	8.67	36.67 ^b^

b—significant difference between groups (*p* < 0.001) adjusted using the Bonferroni method.

**Table 5:** Comparison of personality traits of acrobats according to their sports class.

	First (N = 14)		Master (N = 16)			
*Variables*	*Mdn*	*M*_*rank*_	*Mdn*	*M*_*rank*_	*U*	*p*
Neuroticism	3.00	22.07	2.00	9.75	20.00	<0.001
Extraversion	7.00	15.29	7.00	15.69	115.00	0.918
Openness to experience	5.50	8.50	7.00	21.63	210.00	<0.001
Agreeableness	6.00	19.86	5.00	11.69	51.00	0.010
Conscientiousness	7.00	8.36	9.00	21.75	212.00	<0.001

*Mdn*—median; *M_rank_*—mean rank; *U*—Mann–Whitney test statistics; *p*—significance level.

## Data Availability

The data associated with this article are not publicly available because they contain information that could compromise the privacy/consent of the research participants.
